# Role of Apoptotic Cell Clearance in Pneumonia and Inflammatory Lung Disease

**DOI:** 10.3390/pathogens10020134

**Published:** 2021-01-29

**Authors:** David Jiao Zheng, Maria Abou Taka, Bryan Heit

**Affiliations:** 1 Department of Microbiology and Immunology, Center for Human Immunology, The University of Western Ontario, London, ON N0M 2N0, Canada; dzheng2022@meds.uwo.ca (D.J.Z.); mabouta2@uwo.ca (M.A.T.); 2 Robarts Research Institute, London, ON N6A 5K8, Canada

**Keywords:** efferocytosis, apoptosis, alveolar macrophages, pneumonia, inflammation, specialized pro-resolving mediators

## Abstract

Pneumonia and inflammatory diseases of the pulmonary system such as chronic obstructive pulmonary disease and asthma continue to cause significant morbidity and mortality globally. While the etiology of these diseases is highly different, they share a number of similarities in the underlying inflammatory processes driving disease pathology. Multiple recent studies have identified failures in efferocytosis—the phagocytic clearance of apoptotic cells—as a common driver of inflammation and tissue destruction in these diseases. Effective efferocytosis has been shown to be important for resolving inflammatory diseases of the lung and the subsequent restoration of normal lung function, while many pneumonia-causing pathogens manipulate the efferocytic system to enhance their growth and avoid immunity. Moreover, some treatments used to manage these patients, such as inhaled corticosteroids for chronic obstructive pulmonary disease and the prevalent use of statins for cardiovascular disease, have been found to beneficially alter efferocytic activity in these patients. In this review, we provide an overview of the efferocytic process and its role in the pathophysiology and resolution of pneumonia and other inflammatory diseases of the lungs, and discuss the utility of existing and emerging therapies for modulating efferocytosis as potential treatments for these diseases.

## 1. Introduction

Apoptosis (programmed cell-death) is a highly organized process that is critical for the removal of excessive cells produced during development, for the normal turnover of cells during tissue maintenance, and for the removal of damaged cells during the resolution of pathological events such as infections and inflammation [1]. Every day over a hundred billion cells undergo apoptosis in the adult human body and are replaced by newly formed cells to promote cellular homeostasis. Despite this high burden, clearance of these cells is highly efficient, leaving few uncleared apoptotic cells that can be detected in vivo, even in tissues such as the thymus, which have high rates of cell turnover [2]. This prompt, efficient, and immunologically silent removal of apoptotic bodies by phagocytes is termed “efferocytosis”, and is necessary to prevent secondary necrosis and the subsequent release of proinflammatory alarmins such as adenosine, and autoantigens, which can potentially induce autoimmunity [3]. Efferocytosis is performed by many cell types—termed “efferocytes”—with macrophages functioning as the primary efferocyte in many tissues, while epithelial cells, monocytes, and endothelial cells exhibit efferocytic activity in some tissues [4]. Phenotypically, efferocytosis shares many similarities with the phagocytosis of pathogens [5]. Apoptotic cells are recognized by cell-surface receptors which induce the engulfment of the apoptotic cell into a plasma membrane-derived vacuole termed an “efferosome”. Similar to pathogen-containing phagosomes, these efferosomes undergo a step-wise maturation pathway that delivers the degradative enzymes required to destroy the apoptotic cell (reviewed in [6]). Efferocytosis and phagocytosis are defined by the nature of the target particle being removed by the phagocyte—apoptotic cells in the case of the former, and foreign particulates in the case of the latter—and share many similarities in their cellular and signaling processes. However, there are also differences between efferocytosis and phagocytosis and, therefore, in this review we will refer to efferocytosis/efferosomes when referring to the clearance of apoptotic cells, and phagocytosis/phagosomes when referring to the clearance of microbes and other foreign particulates [3,7,8].

Defects in efferocytosis are known to contribute to many inflammatory and infectious diseases, including lung diseases such as pneumonia, chronic obstructive pulmonary disease (COPD), and cystic fibrosis [9,10]. One of the first investigations into the role of efferocytosis in human pneumonia patients determined that the efferocytic capacity of the patients’ alveolar macrophages correlated more strongly with the recovery of normal lung function than did other markers of disease severity, identifying defective efferocytosis as a contributing factor to the sustained lung injury experienced by some pneumonia patients [11]. Although efferocytosis can be enhanced using existing therapeutics such as glucocorticoids, the use of these agents during infections is controversial as the immunosuppressive action of these drugs impedes some aspects of host defense [12,13,14]. Furthermore, despite the conserved nature of efferocytosis, some of its molecular mechanisms are not fully understood. In this review, we provide an update on the mechanisms and current knowledge gaps of efferocytosis, explore the relationship between efferocytosis and pneumonia, and review the different strategies employed by pneumonia-causing pathogens to influence the efferocytic process to promote their growth. Elucidating the relationship between efferocytosis and pneumonia will inform future therapeutic efforts for manipulating efferocytosis to improve patient outcomes.

## 2. Overview of Apoptosis

Apoptosis, the programmed death of cells, continually occurs under physiological conditions and serves to uphold the homeostasis of tissues through removing damaged or unneeded cells [1,15]. The induction of apoptosis can occur via two different signaling pathways—an intrinsic (mitochondrial) pathway activated by cell stresses including DNA damage and erroneous protein folding in the ER, and an extrinsic pathway activated by the ligation of cell-surface death receptors by ligands expressed on immune cells (reviewed in [16,17]). Apoptosis is coordinated by cysteine aspartic proteases (caspases) which, when activated from their inactive procaspase zymogen forms, cleave various proteins at aspartic acid residues in a cascade leading to the controlled disassembly of intracellular components [18]. Caspases involved in apoptosis are broadly subclassified as initiator caspases (caspase-2, -8, -9 and -10), or executioner caspases (caspase-3, -6 and -7). Initiator caspases are activated by both the intrinsic and extrinsic cell death pathways. In the intrinsic pathway, cell stress pathways inactivate anti-apoptotic proteins, allowing pro-apoptotic Bcl-family proteins to converge on the mitochondria [19,20]. Here, these proteins induce the release of cytochrome C through the opening of the mitochondrial permeability transition pore [21,22]. In the cytosol, cytochrome C complexes with inactive Apoptotic Protease Activating Factor 1 (APAF-1) monomers, inducing the binding of ATP by APAF-1 and the assembly of APAF-1 into a heptamer. Each APAF-1 monomer in this complex, termed the apoptosome, recruits a single molecule of procaspase-9, with this clustering of procaspase-9 allowing cleavage of the pro-domain from neighboring procaspase-9 proteins, thus activating the caspase-9 molecules within the apoptosome [23]. Active caspase-9 in turn cleaves and activates the executioner caspase, caspase-3 [24]. The extrinsic (death receptor) pathway is stimulated by extracellular ligands produced by immune cells that bind to the corresponding death receptors on the dying cells [25,26,27]. The two main receptor-mediated reactions of extrinsic apoptosis are fatty acid synthase ligand and receptor (FasL/FasR) and tumor necrosis factor and receptor (TNF-α/TNFR). Binding of a death ligand with its corresponding death receptor recruits multiple procaspase-8 monomers, which then auto-activate [28]. Active caspase-8 cleaves Bid to form tBid, with both active caspase-8 and tBid recruited to the small amount of charged cardiolipin present on the cytosolic face of the outer mitochondrial membrane. tBid then induces the translocation of additional cardiolipin to the cytosolic facet of the mitochondrial membrane, increasing the surface charge of the mitochondria. This increase in surface charge recruits additional pro-apoptotic proteins and cationic signaling molecules, which ultimately activates the mitochondrial permeability transition pore and initiates apoptosome formation [29,30]. It is not known at this time whether this increase in mitochondrial surface charge occurs during activation of the intrinsic pathway. This activation of the mitochondrial permeability transition pore is not required in all cell types, with direct activation of procaspase-3 by caspase-8-mediated cleavage sufficient to induce apoptosis in some cells [31].

Both the intrinsic and extrinsic pathways converge on the activation of executioner caspases. Once active, these caspases cleave a variety of intracellular targets [18]. Some of these produce active forms of the cleaved proteins, which aid in the disassembly of the cell—e.g., myosin cleavage promotes membrane blebbing [32]—with the activity of these cleaved targets leading to the morphological hallmarks of apoptosis including DNA fragmentation, destruction of cytoskeletal elements, release of ligands recognized by efferocytes, and ultimately, cell death [33,34,35]. After this processing by executioner caspases, the apoptotic cell fragments condense into subcellular membrane-bound vesicles, known as apoptotic bodies, which contain degraded proteins, DNA fragments, and other cellular contents. The final step of apoptosis is efferocytosis, which removes apoptotic bodies prior to the release of intracellular components—thereby reducing the risk of collateral damage to neighboring cells. Efferocytosis (“to bring to the grave” [36]) keeps tissues clear of apoptotic corpses. Efferocytic defects lead to the persistence of apoptotic cells that eventually succumb to secondary necrosis, wherein they lose their membrane integrity via an autolytic process [8,37,38].

The trigger for this transition from the execution phase of apoptosis to secondary necrosis is thought to be ATP depletion in late apoptosis, brought on by a loss of cellular energetics due to mitochondrial dysfunction [39,40,41]. In secondary necrosis, ATP depletion occurs concurrently with ROS production and elevated cytosolic Ca^2+^, resulting in the activation of non-caspase proteases and other enzymes, ultimately rupturing the plasma membrane [42,43,44,45]. The pathogenic consequences of secondary necrosis can be divided into two types: First, membrane rupture results in leakage of cytotoxic pro-inflammatory and immunogenic molecules called damage-associated molecular patterns (DAMPs) or alarmins. These DAMPs include ATP and HMGB1, which are released from the cytosol (ATP) and nucleus (HMGB1) when the membrane integrity of apoptotic cells is lost. These DAMPs can also be released during lytic forms of cell death (e.g., necrosis and pyroptosis, discussed later in this review), and exert their pro-inflammatory effect by activating innate immune cells and inducing tissue injury [46]. Second, autoantigens released after membrane rupture can be taken up by antigen-presenting cells such as dendritic cells and be presented to autoreactive CD4^+^ T cells [37,47]. Activation of autoreactive CD4^+^ T cells can then drive the onset of autoimmune disease, including the activation of B cells to produce autoantibodies, and the activation of cytotoxic CD8^+^ T cells, which together facilitate an autoimmune response. Moreover, in the case of infection, failed efferocytosis may promote the release of the pathogen and infection of neighboring cells [48,49]. Thus, effective efferocytosis is crucial in preventing these immunogenic outcomes.

## 3. Mechanisms of Efferocytosis

While being separate processes, efferocytosis and phagocytosis share many mechanistic similarities. The term “phagocytosis” refers to the receptor-mediated ingestion of large (>0.5 μm) foreign particles into a plasma membrane-derived vesicle known as a phagosome (reviewed in [50]), and shares many of the same uptake and degradative mechanisms as efferocytosis. In most cases, phagocytosis and efferocytosis are performed by professional phagocytic cells such as macrophages, neutrophils, monocytes and dendritic cells—although nonprofessional “neighboring” phagocytes such as epithelial cells, fibroblasts and endothelial cells function as efferocytes in some tissues [51]. Efferocytosis is dependent on several signaling processes that together divide efferocytosis into four sequential steps: “find-me” signals released by apoptotic cells which recruit phagocytes, “eat-me” signals on the apoptotic cell surface which allow for receptor-mediated recognition and engulfment of the dying cell, efferosome formation and maturation that degrades the engulfed material, and anti-inflammatory post-engulfment signaling [52,53].

### 3.1. Release of “Find-Me” Signals by Apoptotic Cells

**Figure 1:**
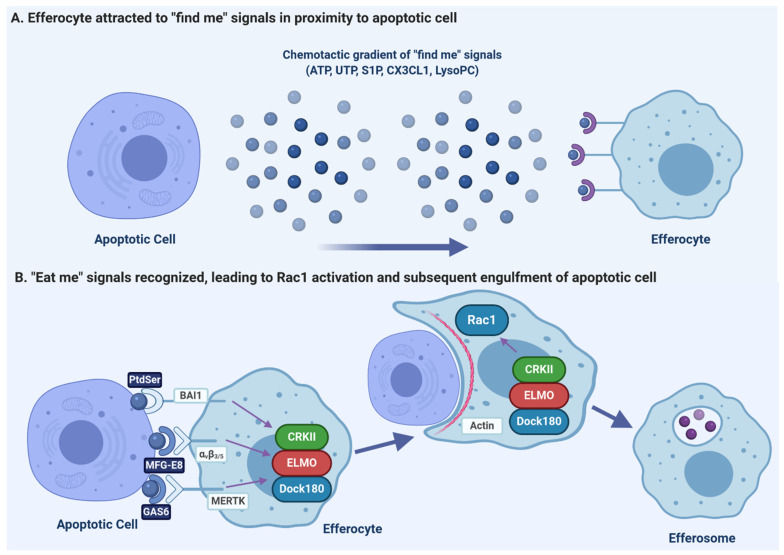
Efferocytosis requires efferocytes to migrate to sites of apoptosis where the apoptotic cells are then engulfed. (**A**) Apoptotic cells release multiple chemoattractants which recruit efferocytes. These include nucleotide-tri-phosphates (ATP, UTP), lipids (S1P, LysoPC), and chemokines (CX3CL1). (**B**) The engulfment of apoptotic cells is primarily driven by the recognition of phosphatidylserine (PtdSer) on the apoptotic cell, either directly by receptors such as BAI1, or via opsonins (MFG-E8 and Gas6) which bridge the PtdSer to efferocytic receptors such as α_v_β_3_ integrin or MERTK. These efferocytic receptors then activate the canonical phagocytic receptor signaling cascade, activating a complex of CRKII, ELMO and DOCK180 which then activates the GTPase Rac1. Rac1 drives the reorganization of the efferocyte actin cytoskeleton such that the efferocyte engulfs the apoptotic cell into a plasma-membrane derived vacuole called the “efferosome”. Figure prepared with BioRender (biorender.com).

For efferocytosis to occur, an efferocyte must first migrate to the apoptotic cell. As in response to pathogens, the migration of efferocytes is controlled by chemoattractant “find-me” signals including chemokines and soluble factors such as nucleotides and lipids which are released into the local environment (Figure 1A). Once released, “find me” signals diffuse and form a chemotactic gradient that allows for the directional migration of efferocytes to the apoptotic cell [54,55,56,57]. Activation of the caspase-3 and -7 results in the release of these soluble mediators [52]. Currently, four find-me signals have been identified, which include CX3CL1 (fractalkine), triphosphate nucleotides (ATP and UTP), sphingosine-1-phosphate (S1P), and lysophosphatidylcholine (LysoPC), which are released at different stages of apoptosis [55,56,58,59]. Elliot et al. found that ATP and UTP release occurs in early apoptosis and induces the directional migration of monocytes towards apoptotic cells in vitro and in vivo, with recognition of these nucleotides occurring through the P2Y_2_ receptor [56]. During apoptosis, caspase-3 and -7 cleave the C-terminal tail of plasma membrane Pannexin-1 (Panx1) channels while the membrane of these cells is still intact, resulting in the opening of the Panx1 channel and release of cytosolic nucleotides into the extracellular milieu where they act as chemoattractants [34]. Another early “find-me” signal released by some early-stage apoptotic cells is CX3CL1, which is released in a caspase-dependent fashion and is recognized by CX3CR1 on efferocytes [58,60]. In addition to acting as a “find me” signal, CX3CL1 also promotes efferocytosis by stimulating expression of MFG-E8 in responding efferocytes, with the MFG-E8 acting as an opsonin that bridges “eat me” signals on apoptotic cells to efferocytic receptors [61].

The find-me signals LysoPC and S1P are lipid chemotactic factors produced by apoptotic cells in later stages of apoptosis [54]. LysoPC is produced from plasma membrane phosphatidylcholine following caspase-3 mediated activation of calcium-independent phospholipase A2. Following its production, LysoPC is released from the cell by ATP-binding cassette transporter C1 and is recognized by the G2A receptor on macrophages [55,62]. Through caspase-1-dependent upregulation of S1P Kinases (SphK) 1 and 2, S1P is generated from sphingosine, released from apoptotic cells, and promotes chemotaxis of macrophages via the S1P receptor (S1PR) [63]. In addition to serving as a “find-me” signal, S1P-induced signaling promotes an anti-inflammatory gene expression phenotype in macrophages, elevating the expression of anti-inflammatory mediators and inhibiting pro-inflammatory mediator production [63]. S1P also directly promotes efferocytosis through inducing the release of erythropoietin (EPO) from apoptotic cells which, via EPO-EPOR signaling, induces upregulation of efferocytic receptors such as MFG-E8 and MERTK by efferocytes [64].

### 3.2. Recognition of Apoptotic Cells via “Eat-Me” Signals

Once attracted by “find-me” signals to the proximity of apoptotic cells, efferocytes recognize specific cell surface ligands on apoptotic cells, termed “eat-me” signals, which differentiate apoptotic cells from neighboring healthy cells displaying “don’t-eat-me” signals such as CD47 [65,66,67]. Efferocytes have various engulfment receptors on their surface that specifically recognize these “eat-me” signals, either directly or via opsonins which act as a “bridge” between the “eat-me” signal and the receptor. During apoptosis, activated executioner caspases cleave different substrates resulting in characteristic morphological changes to the apoptotic cell that allow for this recognition. While a variety of “eat-me” ligands have been studied including cell-surface calreticulin, altered glycosylation patterns of ICAM-1, oxidized lipids, and C1q, the most characteristic and ubiquitously seen marker of apoptosis is exposure of phosphatidylserine (PtdSer) on the outer leaflet of the plasma membrane [68,69,70,71,72,73].

In eukaryotic cells, phospholipids are asymmetrically distributed between the inner and outer leaflets of the plasma membrane [30,74]. In healthy cells, PtdSer is confined to the inner cytoplasmic leaflet by the activity of ATP-dependent flippase enzymes which shuttle PtdSer from the outer to the inner leaflet [75,76]. During apoptosis, PtdSer exposure on the apoptotic cell surface is promoted by the action of two caspase-dependent processes: caspases 3/7 inactivate the flippases ATP11A and ATP11C, and simultaneously activates Xkr8 scramblase, resulting in PtdSer translocation to the outer leaflet [77,78,79]. Once exposed, PtdSer is recognized by many receptors expressed by efferocytes (Figure 1B). Receptors that bind directly to PtdSer include members of the T cell immunoglobulin mucin (TIM) receptor family such as TIM-1, TIM-3, and TIM-4 [80], brain angiogenesis inhibitor 1 (BAI1) [81], stabilin 2 [82], members of the CD300 family [83], and the receptor for advanced glycation end products (RAGE) [84]. Other efferocytic receptors indirectly recognize PtdSer via soluble opsonins such as sCD93 [85], MFG-E8 [86], CCN1 [87], GAS6 [88], and Protein S [88]. After binding to the “eat-me” signals on the apoptotic cell surface, sCD93 is recognized by α_x_β_2_ integrin [85], MFG-E8 and CCN1 are recognized by α_v_β_3/5_ integrins [86,87], while GAS6 and ProS1 are recognized by the TAM (Tyro3, Axl, MERTK) tyrosine kinase family of receptors [88,89,90]. In addition to facilitating the recognition of apoptotic cells by phagocytes, PtdSer also triggers an anti-inflammatory response consisting of secretion of interleukin (IL)-10, and transforming growth factor β (TGFβ), and inhibits the expression of pro-inflammatory cytokines such as TNFα and IL-1β [91,92].

In some instances, healthy cells may have a high amount of PtdSer or other “eat-me” signals on their surface but do not undergo efferocytosis [93,94]. These healthy cells are protected from inappropriate efferocytic uptake by the presence of surface proteins, termed “don’t-eat-me” signals, which bind to receptors on efferocytes to prevent internalization. These “don’t-eat-me” signals include CD47, CD31, and CD24 [66,95,96]. CD47 is recognized by the signal-regulatory protein-α (SIRPα), CD24 is recognized by SIGLEC10, and CD31 prevents engulfment through homotypic interactions with CD31 on efferocytes [67]. In the case of CD47, the most well-studied “don’t-eat-me” signal, the CD47-SIRPα interaction triggers a dephosphorylation cascade which antagonizes efferocytic receptor signaling, ultimately blocking the myosin II and Rac1 activity required for the cytoskeletal rearrangement that mediates apoptotic cell engulfment [66]. As such, any cell that undergoes efferocytosis must first lose their “don’t-eat-me” signals. Dying cells have a reduced expression of CD31 and CD47, thus reducing the inhibitory effect of these “don’t-eat-me” signals [97]. How these “don’t-eat-me” signals are regulated during apoptosis is unclear, although the decrease in CD31 and CD47 is caspase-mediated [97].

The receptors and signaling pathways regulating efferocytosis in response to other “eat me” signals are not as well understood as the receptors and signaling mediated by PtdSer-recognizing receptors. Recognition of oxidized lipids occurs via scavenger receptors such as CD36 and SR-B1 [72,98]. ICAM-1 is recognized by β_2_ integrins [99], while calreticulin is a ligand for C1q and/or LPR [73,100]. Other ligands likely exist—for example, we recently demonstrated that sCD93 acts as an opsonin between apoptotic cells and α_x_β_2_ integrin on the efferocyte, but while we were able to demonstrate that this ligand was not PtdSer, the identity of sCD93’s ligand remains elusive [85]. CD36 and β_2_ integrins utilize variants of the canonical phagocytic signaling pathway, discussed below [101,102], while the efferocytic signaling pathways induced by C1q and LPR are unclear.

### 3.3. Receptor Signaling and Internalization

Once “eat-me” signals are recognized, signal transduction pathways are activated which reorganize the efferocyte actin cytoskeleton at the site of apoptotic cell contact, driving the engulfment of the apoptotic cell (reviewed in [5]). While there are many efferocytic receptors, they appear to utilize the same signaling pathway as canonical pathogen-recognizing phagocytic receptors. This pathway is initiated by two receptor-proximal signals—Src-family kinases and formation of the second messenger phosphatidylinositol(3,4,5)*tris*phosphate (PIP_3_) by PI-3-kinase (PI3K) [103,104,105]. These pathways activate a host of downstream effectors, converging on the activation of the RHO family GTPase Rac1 [103]. The activation of Rac1 requires the formation of a functional Rac1 guanine exchange factor complex, usually composed of a complex of DOCK180 and ELMO1, on the cell membrane (Figure 1B, [81,106,107]). DOCK180 is recruited to the membrane by PIP_3_, after which it binds to ELMO1 via an SH3 domain; this complex forms a bipartite guanine-nucleotide exchange factor which then activates Rac1 [106]. Efferocytic receptors which use the CrkII-ELMO-Dock180 Rac1-activating pathway include integrin α_v_β_3/5_, MERTK, and BAI1 [81,108,109]. Activation of Rac1 by the ELMO1/DOCK180 complex reorganizes the cortical actin cytoskeleton through Arp2/3 induced actin polymerization [82,105,107]. This polymerization drives extensions of the plasma membrane around the apoptotic cell, first forming a cup-like structure, which over time completely envelops the apoptotic cell. This membrane extension can require the addition of membrane to the forming cup, which is delivered via the focal exocytosis of ER- and endosome-derived vesicles, with these fusion events mediated by Arf6, Rab13 and Rab35 (reviewed in [6]). Fusion of the enveloping membrane and closure of the phagocytic cup forms a discrete cytosolic vacuole, termed the “efferosome”, containing the apoptotic cell. This process appears to be identical to the engulfment process of phagocytosis, with the same canonical signaling pathway activated by both efferocytic and phagocytic receptors and driving an engulfment process relying on the same cytoskeletal rearrangements and Rab-mediated membrane delivery.

### 3.4. Efferosome Maturation

Once the apoptotic cell is internalized, the resulting efferosome proceeds through a tightly regulated maturation process that degrades the internalized contents through the sequential fusion of the efferosome with early endosomes, late endosomes and ultimately lysosomes. This delivers hydrolytic enzymes and vacuolar ATPases, which produce an increasingly acidic luminal environment that bestows the efferosome with potent degradative capabilities. Each stage of this process is facilitated by the action of Rab GTPases (reviewed in detail in [6]).

**Figure 2:**
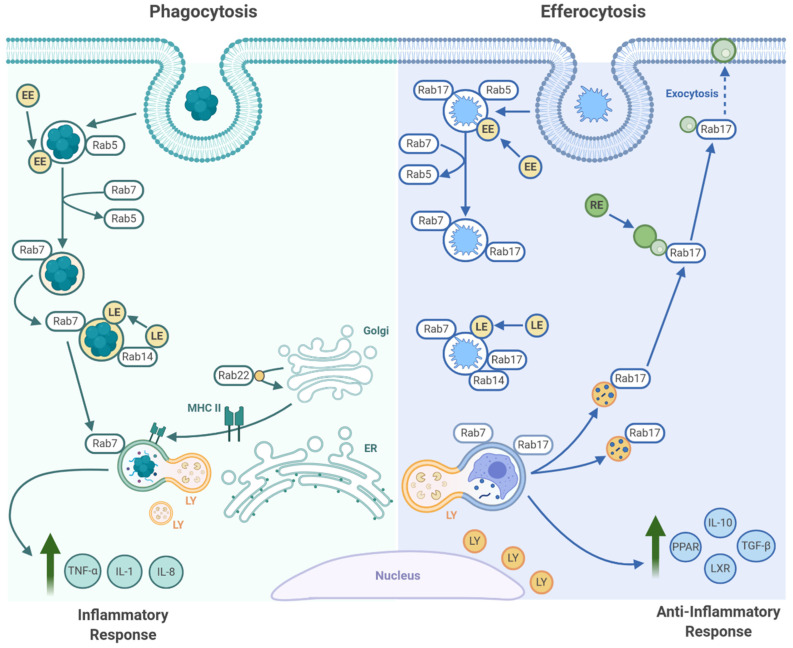
Maturation of pathogen-containing phagosomes and apoptotic cell-containing efferosomes. Following pathogen phagocytosis (left), the microbes are contained within a membrane-bound phagosome, which recruits the GTPase Rab5. Rab5 then mediates fusion of the phagosome with early endosomes (EE), after which Rab5 is exchanged for Rab7 on the phagosome surface. Rab7 then mediates fusion of the phagosome with late endosomes (LE) and lysosomes (LY), thereby killing and degrading the microbe. Microbe-recognizing pattern recognition receptors induce the expression of inflammatory cytokines and drive macrophage polarization towards a more inflammatory phenotype. In some cell types (macrophages and dendritic cells), this inflammatory signaling also induces MHC II expression. MHC II is then trafficked to the phagosomes, where it is loaded with microbe-derived antigens that can then be presented to T cells. The initial stages of efferocytosis are similar to phagocytosis in that Rab5 and Rab7 drive fusion of the efferosome with endosomes and lysosomes, but differs in that Rab17 is also recruited to the efferosome. At the later stages of maturation, Rab17 mediates the fragmentation of the efferosome and the transport of these efferosome fragments to the recycling endosomal system (RE), where these materials are absorbed. Efferocytic receptors and efferosome-derived materials activate transcription factors that induce the expression of anti-inflammatory cytokines and mediate the metabolic reprogramming of the efferocyte to enable the engulfment of additional apoptotic cells. Figure prepared with BioRender (biorender.com).

The first maturation step after engulfment is fusion of the nascent efferosome with early endosomes (Figure 2). This is driven by the Rab GTPase Rab5, which is activated on the early efferosome by the GEF Rabex-5 [110]. Once activated, Rab5 recruits and activates several Rab5 effector proteins, including the type III PI3K Vps34, which catalyzes the generation of the signaling lipid phosphatidylinositol-3-phosphate (PI3P) [111]. The accumulation of PI3P on the cytosolic leaflet of the early efferosome recruits and activates other Rab5 effectors, such as endosomal early antigen 1 (EEA1) and rabenosyn-5, which together trigger the formation of the class C COre Vacuole/Endosome Tether complex (CORVET) on the efferosome membrane [111,112,113,114]. This complex bridges active Rab5 on the efferosome to Rab5 on early endosomes, thereby driving the SNARE-dependent fusion of the efferosome with early endosomes. This begins the delivery of the degradative enzymes which will ultimately destroy the apoptotic cell [115,116].

Approximately five minutes after engulfment, the early efferosome transitions to a late efferosome marked by an exchange of Rab5 for Rab7 (Figure 2, [117]). A complex of Monensin and Brefeldin A Hypersensitive 1a/b (Mon1a/b) and Sensitive to Caffeine Ca^2+^ and Zn^2+^ (Ccz-1) promotes the inactivation and release of Rab5. In parallel, the Mon1a/b/Ccz-1 complex recruits and activates Rab7 [118,119]. Once activated, Rab7 recruits a set of effectors different from those recruited by Rab5. This includes Rab-interacting lysosomal protein (RILP) and oxysterol-binding protein related-protein 1 (ORPL1), which interact with dynein/dynactin to transport the late efferosome to the perinuclear region of the cells where fusion between the efferosome and lysosomes occurs [118,120,121]. Through these effectors, Rab7 also promotes recruitment of the HOmotypic fusion and Protein Sorting complex (HOPS), a large multimeric tethering complex necessary to dock and fuse the efferosome with Rab7-bearing late endosomes and lysosomes (reviewed in [114]). This complex bridges Rab7 on the efferosome with Rab7 on late endosomes and lysosomes, thus enabling SNARE-mediated fusion of late endosomes and lysosomes with the efferosome. These fusion events deliver V-ATPases, which lowers the luminal pH to 5.0 or lower, and hydrolytic enzymes including nucleases, cathepsins, and lipases, to the efferosome [122,123]. The high acidity of the luminal environment produced by the V-ATPases not only serves to facilitate degradation of the apoptotic cell debris but also works synergistically to activate the hydrolases acquired from the lysosomes. Together, the hydrolytic enzymes and acidic environment, along with production of oxygen radicals by NADPH oxidase, results in the complete degradation of the internalized apoptotic cell [124,125].

During pathogen phagocytosis, the highly acidic compartment that forms after phagosome-lysosome fusion (the phagolysosome [126,127]) matures further, forming into an MHC class II loading compartment (MIIC) [128,129,130]. Here, pathogen-derived antigens are loaded on to MHC II, and then exported to the cell surface for presentation to antigen-specific CD4^+^ T cells (reviewed in [129]). However, this process does not occur following efferocytosis, as efferocytosis is anti-inflammatory and non-immunogenic. Our group recently determined that this key difference in pathogen phagocytosis versus apoptotic cell efferocytosis is driven by the recruitment of Rab17 onto the early efferosome [131,132]. Rab17 persists on the efferosome throughout maturation, where following lysosome fusion and degradation of the apoptotic cell, Rab17 mediates trafficking of the efferosome from the perinuclear region to the cell periphery [133]. Here, the apoptotic cell contents are shuttled to the recycling endosome, where the degraded materials are thought to either be absorbed by the efferocyte or expelled via exocytosis. This may act to enhance the recovery of amino acids, lipids, sugars and proteins from the apoptotic cell, while simultaneously limiting immunogenic loading of autoantigens onto MHC II [132,133]. While the specific mechanism which allows Rab17 to be persistently recruited to the efferosome remains unknown, it is established that MIIC formation requires TLR signaling, and Rab17 knockdown enables MHC II recruitment to efferosomes, suggesting that Rab17 may antagonize MIIC formation and is displaced from phagosomes via a TLR-dependent signaling mechanism [132,134,135]. It is currently unknown how Rab17 prevents MHC II recruitment to the phagosome, with this process currently being under investigation in our lab.

## 4. Efferocyte Metabolism, Polarization, and Inflammation

While the normal outcome of phagosome maturation is the initiation of inflammatory signaling, efferosome maturation is characterized by the opposite—the secretion of anti-inflammatory and pro-tissue healing factors. This includes upregulation of anti-inflammatory cytokines including TGF-β, IL-10, and release of lipid mediators including prostaglandin E2 and I2 (PGE_2_, PGI_2_) [91,136]. In addition, efferocytosis also inhibits secretion of pro-inflammatory cytokines, such as TNF-α, IL-1, and IL-8, through suppression of the nuclear factor-κ B (NF-ĸB) transcription [91,137]. Further anti-inflammatory influence comes from activation of the TAM family of efferocytic receptors such as MERTK, which upregulate the expression of suppressor of cytokine signaling-1 and -3 (SOCS1 and SOCS3), which attenuate inflammatory responses by inhibiting STAT-dependent transcription [138,139].

### 4.1. Efferocyte Metabolism and Polarization

Changes in efferocyte behavior following efferocytosis is not limited to the production of anti-inflammatory mediators, and indeed, following efferocytosis, these cells undergo large-scale metabolic and transcriptional reprogramming. Efferocytic receptor signaling induces the upregulation of liver X receptor (LXR) and peroxisome proliferator-activated receptor (PPAR) nuclear receptors [140,141,142]. LXR and PPAR receptors are generally co-expressed in phagocytic cells under basal conditions, although when singly-expressed, activation of one receptor induces the expression of the other [143,144]. LXR and PPAR receptors bind to many of the same ligands—mostly lipid-derived metabolites—with activation of both leading to the preferential formation of heterodimers over homodimers. Once dimerized, the receptors bind to their target gene promotors, with the homodimeric and heterodimeric forms recognizing the same DNA sequence [145]. Signaling of the heterodimers are best understood, as these are most often found in efferocytes, where they enhance the expression of efferocytic receptors and cholesterol exporters. These receptors can also suppress gene expression, for example inhibiting pro-inflammatory gene transcription through suppressing the transcription of STAT-3 [146,147,148,149]. While heterodimer-mediated transcription seems to be most common in efferocytes, competition between homodimers for DNA binding sites may also have an important role in determining the response of efferocytes to apoptotic cell engulfment. For example, active LXR can displace PPAR homodimers, thereby preventing the PPAR-mediated inhibition of fatty acid β-oxidation [150]. The role of these receptors in pathogen phagocytosis are less well understood. PPAR activation with synthetic ligands enhances the expression of macrophage NADPH oxidase, myeloperoxidase, and upregulates CD36 which, in addition to acting as an efferocytic receptor, can act as a phagocytic receptor for eukaryotic pathogens such as malaria [151,152,153]. The role of LXRs in phagocytosis appears to be context dependent. LXR agonists in the absence of other inflammatory stimuli enhance phagocyte responsiveness to pathogens through upregulating TLR receptors and reactive oxygen species production, but the effect of LXR signaling rapidly becomes strongly anti-inflammatory upon exposure of the phagocyte to inflammatory cytokines or pathogens [154,155].

These changes in efferocyte metabolism are critical for efficient and on-going efferocytosis. The high metabolic burden placed on efferocytes—a doubling or more of their lipid and sterol content every time an apoptotic cell is engulfed—requires a significant alteration to the efferocytes’ metabolism. This includes upregulation of the mitochondrial electron transport chain and enzymes for lipid β-oxidation, allowing efferocytes to derive most of their energy from lipolysis, while also reducing their lipid content [156]. In parallel, macrophages upregulate the cholesterol exporters ABCA1 and ABCG1, enhancing their export of cholesterol to HDL [157,158]. This enhanced metabolic capacity is required for efferocytes to engage in multiple rounds of efferocytosis. Indeed, inhibition of these pathways limits the ability of efferocytes to engulf multiple apoptotic cells, as the cellular stresses from the buildup of apoptotic cell-derived lipids and sterols prevents further rounds of efferocytosis [159,160]. This phenomenon is not limited to in vitro studies, and indeed, is partially responsible for the pathology of atherosclerosis. In atherosclerosis, the accumulation of lipids and sterols in subvascular macrophages induces the expression of the transcription factor GATA2. GATA2 then suppresses the transcription of efferocytic receptors (e.g., α_x_β_2_), efferocytic signaling molecules (e.g., Src-family kinases), regulators of efferosome maturation (e.g., Rab7), and hydrolytic enzymes required for degradation of apoptotic cells (e.g., vacuolar ATPase, [161]). The loss of these efferocytic regulators then limits the clearance of apoptotic cells, allowing for the accumulation of dead, cholesterol-laden macrophages that comprise the bulk of atherosclerotic plaques.

In macrophages, this anti-inflammatory and metabolic reprogramming results in a unique polarization state different from the classically-defined inflammatory (M1) and alternatively activated (M2) polarization states. Indeed, transcriptomic analysis of tissue macrophages fails to find cells with gene expression patterns similar to in vitro polarized M0/M1/M2 macrophages and, rather, the transcriptomic profile of tissue-resident macrophages varies greatly between tissues [162]. However, while tissue resident macrophages vary greatly between tissues, the subset of macrophages in each tissue specialized for efferocytosis share a common transcriptional “core” characterized by expression of the scavenger receptors CD206 and CD163 (recognizing mannose and hemoglobin-haptoglobin, respectively) and the efferocytic receptors Tim-4 and MERTK. This “core” signature was not found in M0/M1/M2 polarized cells, nor was it found in non-efferocytic macrophages from the same tissues [162]. It is unclear if a similar transcriptional pattern emerges when non-professional efferocytes engulf apoptotic cells.

### 4.2. Specialized Pro-Resolving Mediators

In responses to engulfing an apoptotic cell, efferocytes produce several anti-inflammatory proteinaceous, gaseous and lipid-based mediators, which act on neighboring cells in a paracrine fashion. In the human respiratory system, this anti-inflammatory response is driven primarily by Specialized Pro-resolving Mediators (SPMs), lipid mediators derived from dietary essential polyunsaturated fatty acids (reviewed in [163,164]). Examples of SPMs include arachidonic acid-derived lipoxins, and omega-3 fatty acid-derived resolvins, maresins, and protectins. SPMs are formed during acute inflammation, when lipid mediator class-switching drives polyunsaturated fatty acid metabolism from the production of pro-inflammatory mediators such as prostaglandins and leukotrienes, to production of pro-resolving SPMs [165,166]. In the lung, SPMs can be synthesized via a number of cell types, with lipoxins being produced by epithelial cells, eosinophils, and monocytes/macrophages [167,168], while resolvins can be produced by endothelial cells and leukocytes [169,170], and maresins by macrophages [171]. SPMs are stereoselective agonists of their cognate G-protein coupled receptors, which are expressed by leukocytes [172]. Consequentially, SPMs can act in both a paracrine and autocrine fashion, and indeed, the induction of SPM synthesis following efferocytosis has been proposed to generate a positive feedback loop that reinforces the anti-inflammatory signaling induced by efferocytic receptors (reviewed in [164]). In the inflamed lung, SPMs decrease leukocyte infiltration while increasing efferocytic clearance of apoptotic leukocytes and increasing the production of anti-microbial peptides [173,174]. While there are multiple classes of SPMs, resolvins are the best understood and have well-established roles in regulating efferocytosis and inflammation. Moreover, exposure to pathogenic bacteria, such as *S. aureus* and *E. coli*, has been shown to stimulate human alveolar macrophages in the lungs to produce SPMs, notably, resolvin D1 (RvD1) and resolvin D2 (RvD2), to mitigate inflammation [175].

ALX/FPR2, ERV, BLT1, DRV1 and DRV2 are high-affinity receptors for resolvins (reviewed in [176]). These receptors are primarily expressed on human airway epithelial cells and on innate immune cells such as macrophages and neutrophils [64,173]. ALX/FPR2 and DRV1 are high-affinity RvD1 receptors, while ERV, BLT1 and DRV2 are high-affinity RvE1 and, potentially, RvE2 receptors [174]. While these receptors bind to different types of resolvins, they share the common anti-inflammatory functions including promoting efferocytosis and bacterial phagocytosis, while limiting neutrophil recruitment and migration. The expression of these high affinity resolvin receptors are regulated by cytokines and epigenetic changes which occur in response to inflammation. While acute inflammation tends to increase the expression of these receptors, chronic airway inflammation suppresses resolvin receptor expression, which in turn, hinders the homeostatic effects of resolvins [174,177].

Furthermore, some patients with bacterial pneumonia will subsequently develop respiratory failure and acute respiratory distress syndrome (ARDS), prolonging inflammatory responses in the lungs [177]. In humans, the underlying cellular mechanisms involved in the development of chronic inflammation in patients with bacterial pneumonia are not completely established. However, murine models of bacterial pneumonia demonstrated that resolvin administration enhances the resolution of inflammation. Specifically, resolvins were shown to promote efferocytosis of apoptotic leukocytes and reduce neutrophil recruitment to the lungs in murine models [178]. Interestingly, human chronic inflammatory diseases of the lung such as asthma, cystic fibrosis and COPD, exhibit a similar defect in SPM activity to patients with acute inflammation from ARDS and respiratory failure following bacterial pneumonia [174]. Notably, the persistent inflammation exhibited in asthma, cystic fibrosis and COPD is due, in part, to insufficient levels of SPMs or their receptors in the airways of these patients [178].

## 5. Alveolar Macrophages and Efferocytosis in the Lung

In the proximal airways, the clearance of debris is overseen by pulmonary mechanisms such as the mucociliary apparatus, while the distal airways and lungs are kept clear by phagocytosis and efferocytosis. In healthy lungs, efferocytosis is predominantly mediated by alveolar macrophages located in the mucus layer and interstitial space, and are the most prevalent alveolar professional phagocytes, making up 95% of cells retrieved by bronchoalveolar lavage [179]. Unlike some macrophage populations, alveolar macrophages are long-lived and have a high capacity for self-renewal [180]. Other alveolar efferocytes include recruited monocytes and dendritic cells, as well as non-professional phagocytes such as epithelial and mesenchymal cells [181]. Together, these cells both maintain homeostasis in the airway and aid in the recovery from tissue injury.

Under homeostatic conditions, few apoptotic cells are found in the airways due to the efficient clearance by alveolar efferocytes [182]. Even during acute lung inflammation, which typically features high levels of immune cell apoptosis, few uncleared apoptotic cells are found within the lung [183]. Alveolar macrophages differ from other tissue-resident macrophages; in particular, alveolar macrophages in the non-inflamed lung have a reduced ability to engulf both efferocytic and phagocytic targets compared to other tissue-resident macrophages from other organs. In addition, alveolar macrophages do not respond in an inflammatory fashion after engulfing single bacteria. This reduced responsiveness is thought to prevent inflammatory damage to the vulnerable alveolus following inhalation of cellular debris, non-pathogenic bacteria, and harmless particulates [179,184,185]. In comparison, most other tissue macrophages will readily engulf efferocytic and/or phagocytic targets in large numbers, and respond with a robust activation of inflammatory genes in response to single bacteria [186,187]. This reduced activity of alveolar macrophages is the result of the local airway microenvironment which influences both the expression pattern of efferocytic receptors and overall efferocytic capacity of alveolar macrophages, and is mediated by both soluble mediators and cell-cell interactions. Type II alveolar epithelial cells in the lower airways secrete pulmonary surfactant fluid containing the lung collectins pulmonary surfactant-associated protein (SP)-A and SP-D, which bathes the alveolar macrophages [188]. SP-A and SP-D act as ligands for the SIRPα receptor on efferocytes which inhibits efferocytosis [180,188]. In addition, SP-A and SP-D and promote an anti-inflammatory state by inhibiting formation of the C1 complex required for activating complement, and by blocking TLR2 and TLR4 interactions also prevents NF-ĸB signaling [189,190,191]. Finally, IL-10 is constitutively expressed by lung epithelial cells [192], thereby producing an intracellular milieu that suppresses alveolar macrophage activity.

This tonic inhibition is also mediated by cell contact-dependent processes through molecules expressed by the airway epithelium. Type II alveolar epithelial cells express CD200, which binds to CD200 receptors on alveolar macrophages to block the MAPK and JNK inflammatory pathways [193]. TGF-β is expressed by lung epithelium, and is tethered to the surface of these cells, providing a contact-dependent activation of the TGF-β receptor [194]. While the mechanisms underlying this decrease in alveolar macrophage suppression remain unclear, research has suggested that the damage to the respiratory epithelium and consequent reduction in exposed epithelial cell ligands may relieve this suppressive effect on macrophages, allowing them to engage in more efficient phagocytosis and efferocytosis. Restoration of the lung epithelium then restores normal expression of IL-10 and CD200, restoring the tonic suppression of alveolar macrophages following the resolution of inflammation [195,196]. In addition, the high concentration of multiple inflammatory signals present during an infectious or inflammatory event transcriptionally suppresses some of the tonic inhibitory pathways. For example, TNF-α suppresses the transcription factors necessary for IL-10 expression [197]. Thus, through a combination of transcriptional regulation and the loss of lung epithelial cells, the tonic inhibitory environment in the lung is downregulated during inflammation, thereby “freeing” alveolar macrophages to take on an inflammatory phenotype.

During pneumonia, alveolar macrophages have been reported to exhibit a significant efferocytic capacity, but it is unclear how this is induced [11]. While alveolar macrophages appear to take on an inflammatory phenotype during events such as infection, a large portion of the macrophages in the inflamed lung are derived from the differentiation of blood-derived monocytes into inflammatory and pro-fibrotic macrophages [198,199]. Moreover, the recent description of a new lung-resident macrophage population challenges the view that alveolar macrophages are the predominant cell type responsible for efferocytosis in the lung [198]. These nerve- and airway-associated macrophages are found in the lung’s interstitium and maintain an anti-inflammatory and pro-efferocytic phenotype even under inflammatory conditions. This may indicate that it is the monocyte-derived or interstitial lung-resident macrophages, and not the airway-resident alveolar macrophages, that are the primary mediators of efferocytosis in the lung.

## 6. Impaired Efferocytosis in Lung Disease

An increased number of uncleared apoptotic cells have been detected in the airways of patients with several chronic inflammatory lung diseases, with alveolar macrophages from these individuals exhibiting defects in efferocytosis. Some evidence suggests that impaired apoptotic cell clearance may be upstream to the sustained lung inflammation of these diseases, and causally contributes to their pathogenesis [200].

### 6.1. Chronic Obstrructive Pulmonary Disorder

COPD is the chronic inflammation of the airways maintained by the continuous accumulation of activated neutrophils, macrophages and T cells, which cause irreversible small-airway obstruction, peribronchial fibrosis, and emphysema. In COPD, the efferocytic uptake of apoptotic bronchial epithelial, immune and endothelial cells by alveolar macrophages in the lungs is significantly reduced, with a corresponding accumulation of apoptotic cells [9,201]. This efferocytic defect was found to be more pronounced in COPD patients who smoke, with cigarette smoking being the number one risk factor for COPD [202]. In addition, the reductions in efferocytic potential were also associated with altered expression of CD31, CD44, and CD91/LRP-1—key proteins involved in apoptotic cell recognition and binding [202]. While the mechanisms underlying these efferocytic defects remain unidentified, potential contributors include alteration of PtdSer-recognizing efferocytic receptors and opsonins [203], increased levels of HMGB1 in the airways [204], and downregulation of SP-D during chronic lung inflammation [205].

### 6.2. Asthma

Asthma is another obstructive pulmonary disease characterized by hyperresponsiveness to airway allergens and subsequent recruitment of eosinophils and chronic inflammation. As with COPD, asthma is also associated with an increased number of uncleared apoptotic epithelial cells [206]. Alveolar macrophages in patients with severe asthma have reduced production of anti-inflammatory prostaglandins which, alongside secondary necrosis, may contribute to the chronicity of inflammation [206]. The role of efferocytosis in the pathogenesis of asthma remains poorly understood, but interestingly, glucocorticoids are known to enhance efferocytosis in asthma patients, suggesting that improved efferocytosis may be partially responsible for the positive effects of glucocorticoids in asthmatic patients [1].

### 6.3. Cystic Fibrosis

Cystic fibrosis (CF) is a genetic disorder caused by a mutation in the CF transmembrane conductance regulator (CFTR) gene. The loss of CFTR, a chloride ion channel, produces thicker respiratory mucous, promotes bacterial colonization, leading to chronic neutrophilic inflammation and impaired mucociliary clearance [207]. Similar to asthma and COPD, CF patients have an accumulation of uncleared apoptotic cells in the airway, primarily uncleared apoptotic neutrophils. These neutrophils release elastase, both during extracellular trap formation and during secondary necrosis, with this elastase reducing efferocytosis through cleavage of efferocytic receptors [10,208,209]. Other potential contributors to this efferocytic defect include increased levels of HMGB1 in the extracellular space [210], and increased RhoA activity through cytokine-mediated decreases in the expression of the RhoA negative regulator FAM13A, and cytokine-induced STAT1-mediated transcription of RhoA itself [211,212].

### 6.4. Lung Cancer

Through its immunosuppressive effects, efferocytosis also plays a role in promoting the tumor microenvironment and tumorigenesis and progression [213]. Macrophages are the primary immune cell found in tumor microenvironments. PtdSer receptors, such as the TAM and TIM family receptors, are expressed on these cells and are frequently stimulated by apoptotic cells within the tumor environment. This triggers the anti-inflammatory signaling pathways downstream of these receptors, leading to the increased production of anti-inflammatory and pro-healing cytokines. This altered inflammatory environment promotes tumor growth and survival, while dampening T cell-mediated tumor immunity (reviewed in [214]). Indeed, efferocytic receptors are often overexpressed directly by cancers cells, including small and non-small cell lung carcinoma, and contribute to drug resistance to antitumor therapies [215].

### 6.5. Community-Aquired Pneumonia

Since efferocytosis is essential for resolution of lung inflammation, it follows that it may be an important factor in the differing outcomes of pneumonia patients. In one prospective cohort study, the recovery of lung function after community acquired pneumonia (CAP) was found to be positively correlated with the rate of efferocytosis exhibited by patients’ alveolar macrophages [11]. Another study using a double-infection mouse model of secondary pneumonia found that, following recovery from primary pneumonia caused by *Escherichia coli*, *Staphylococcus aureus*, or the influenza A virus, alveolar macrophages have a severely impaired phagocytic capacity associated with an increased susceptibility to secondary infection upon re-exposure to causative organisms [210]. Renewal of the alveolar macrophage population with this functional impairment was from locally renewing tissue-resident macrophages which had undergone epigenetic reprogramming. This epigenetic reprogramming was induced by sustained secondary inflammatory mediator signaling and enhanced SIRPα expression and activity from the post-pneumonia lung environment, rather than bacterial pathogen products from primary pneumonia [210]. Upon reinfection and induction of pneumonia, the reprogrammed alveolar macrophages had reduced phagocytic capacity. While inhibition of SIRPα-CD47 interactions may have potentially therapeutic application for preventing hospital-acquired pneumonia, further functional analyses of alveolar macrophages in different models is warranted. Unlike COPD, asthma and CF, pneumonia is driven entirely by infection of the lung. As such, some of the pathology of pneumonia may be a result of pathogen manipulation of the efferocytic system.

## 7. Pathogen Manipulation of Efferocytosis

Through the clearance of apoptotic cells, efferocytosis prevents secondary necrosis and induces an anti-inflammatory state through the release of anti-inflammatory mediators PGE_2_, IL-10 and TGF-β. This ultimately promotes the resolution of inflammation and healing following an infection. In addition, induction of apoptosis and subsequent apoptotic cell engulfment is critical for destruction of certain intracellular pathogens, such as *Mycobacterium tuberculosis*, which would otherwise disseminate freely following the death of infected cells [48]. While it is tempting to consider efferocytosis as a potential therapeutic target for enhancing the clearance of pneumonia-causing pathogens and improving lung function following pneumonia, pathogens engage in species-specific manipulation of cell death and efferocytic pathways. Therefore, it is important to understand how pathogens manipulate cell death and efferocytic pathways, and efferocytosis targeting therapies will likely need to be employed in an organism-specific manner.

### 7.1. Subverting Efferocytosis

Although efferocytosis can have an antimicrobial effect and clear pathogens by removing apoptotic phagocytes, some pathogens have developed strategies to avoid efferocytosis through the action of secreted virulence effectors that alter host cell processes.

#### 7.1.1. *Streptococcus pneumoniae*: Inducing Apoptosis to Limit Microbicidal Activity

While the anti-inflammatory state induced by efferocytosis effectively maintains immune homeostasis, it may also inhibit the mounting of an antimicrobial immune response and increase the susceptibility to other infections. Pathogens such as *Streptococcus pneumoniae* induce apoptosis, with the subsequent efferocytosis of the infected cells and the PGE_2_ production this induces in alveolar macrophages, inhibiting bacterial killing through reduced production of microbicidal H_2_O_2_ in the efferosome. This inhibition occurs through PGE_2_ receptors EP-2 and EP-4, which inhibit H_2_O_2_ production via cAMP and PKA signaling [216,217]. While these studies did not identify the mechanism by which *S. pneumoniae* induced cAMP/PKA activity inhibits H_2_O_2_ production, PGE_2_ has a similar effect on responses to *Klebsiella pneumoniae*, where PKA activation blocks translocation of the p47phox subunit of NADPH oxidase to the phagosome, thereby preventing formation of active NADPH oxidase and the resulting generation of superoxide and H_2_O_2_ [218].

#### 7.1.2. *Legionella*, *Salmonella* and Tuberculosis: Inhibiting Apoptosis

A strategy employed by pathogens to avoiding efferocytosis is inhibiting apoptosis. To inhibit macrophage apoptosis, *Legionella pneumophila* secretes LegK1—a bacterial effector—into the cytosol of the infected macrophage. This activates NF-κB mediated transcription in the infected cell, increasing the expression of the anti-apoptotic gene plasminogen activator inhibitor-2 which then prevents apoptosis of the infected cell [219]. *Salmonella enterica* secretes its effector SopB into the cytosol of infected epithelial cells, where it inhibits apoptosis by activating the anti-apoptotic kinase Akt [220]. One pathway contributing to survival of some virulent forms of *M. tuberculosis* functions by inhibiting macrophage apoptosis through activity of its NADH oxidoreductase NDH-1, a component of the mycobacteria electron transport chain which directly neutralizes ROS. By eliminating the ROS produced by infected macrophages, NDH-1 prevents ROS-induced macrophage apoptosis [221,222]. For these pathogens, inhibiting apoptosis both prolongs their growth period in the infected cell and leads to dissemination via the eventual necrotic lysis of the infected cell.

#### 7.1.3. *Klebsiella pneumoniae*: Manipulating Cell Death Pathways

**Figure 3:**
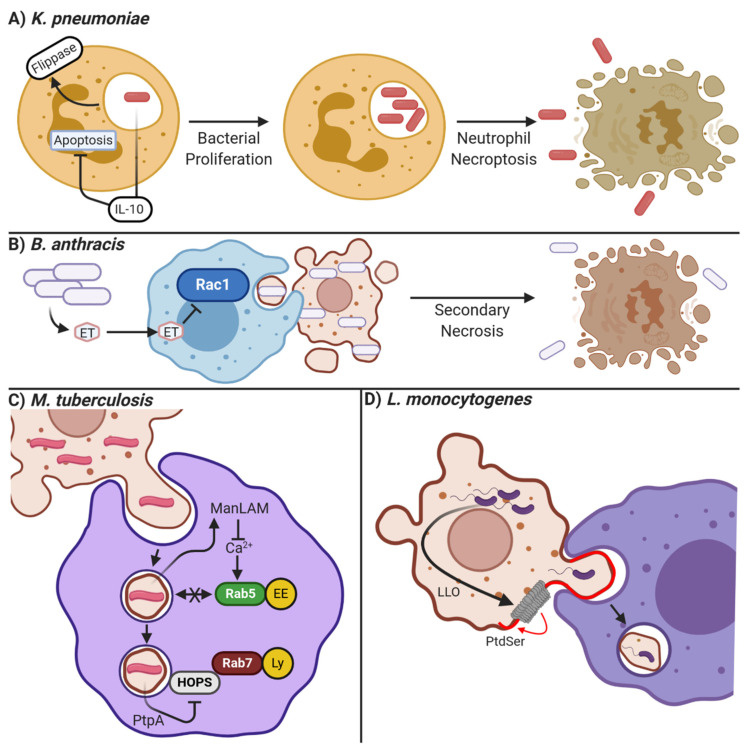
Mechanisms of bacterial manipulation of apoptosis and efferocytosis. (**A**) *K. pneumonia* suppresses neutrophil apoptosis through inducing the expression of IL-10, and efferocytosis through activation of membrane flippases, which prevent PtdSer from accumulating on the cell surface. This allows the bacteria to reproduce in the neutrophil, eventually escaping via lytic necroptotic cell death. (**B**) *B. anthracis* secretes edema toxin (ET) which can enter neighboring cells and inhibit Rac1-mediated cytoskeletal reorganization. This prevents the efferocytosis of *B. anthracis*-infected apoptotic cells, allowing for additional bacteria growth and their eventual release following secondary necrosis of the infected cell. (**C**) *M. tuberculosis* is often engulfed by macrophages inside of apoptotic bodies derived from infected cells. *M. tuberculosis* can then proliferate within the efferosome by ManLAM-mediated inhibition of calcium-dependent fusion of early endosomes (EE) to the efferosome, and PtpA mediated inactivation of the HOPS complex required for Rab7-mediated fusion of lysosomes (LS). (**D**) *L. monocytogenes* releases a pore-forming toxin LLO (grey ring) which induces blebbing and PtdSer externalization on infected cells. Neighboring macrophages engulf these blebs, thereby becoming infected with *L. monocytogenes*. Figure prepared with BioRender (biorender.com).

Other pathogens avoid efferocytosis by manipulating the cell death process itself. *Klebsiella pneumoniae*, a common cause of nosocomial pneumonia, utilizes multiple mechanisms to manipulate cell death and prevents its clearance by efferocytosis. *K. pneumoniae* impairs the efferocytosis of infected neutrophils by increasing the activity of flippases, preventing PtdSer externalization and therefore recognition by efferocytes (Figure 3A [223]). In some *K. pneumoniae* strains, this is accomplished by a suppression of signaling via the extrinsic apoptosis pathway, leading infected neutrophils to proceed down the necroptotic cell death pathway. Necroptosis occurs when the extrinsic apoptosis pathway is activated under conditions where initiator caspase-8 (and therefore apoptosis) cannot be activated. This results in activation of RIPK1 and RIPK3, which induce the polymerization of MLKL, thus forming pores in the plasma membrane and mitochondria that lyse the cell (reviewed in [224]). Thus, necroptosis spills the bacterium into the interstitium rather than allowing its uptake and clearance through efferocytosis [223]. In addition to activating necroptosis, *K. pneumoniae* also inhibits the pyroptotic cell death pathway to limit inflammation. Pyroptosis is an inflammatory form of lytic cell death that is triggered by the presence of cytosolic bacterial products. Pyroptosis is initiated by bacterial product-mediated activation of the inflammasome—a cytosolic bacterial sensor—which then cleaves and activates caspases-1,-4 and -5 [225]. Additionally, caspases 4 and 5 can be directly activated by LPS-mediated polymerization [226]. Unlike other caspases, these caspases do not induce apoptosis and rather mediate the cleavage-induced secretion of the inflammatory cytokines IL-1β and IL-18 [227]. This secretion involves two caspase-mediated events: 1) the cleavage of pro- IL-1β and pro-IL-18 into their active form, and 2) cleavage of GSDMN, which in its cleaved form polymerizes into a pore in the plasma membrane through which IL-1β and IL-18 are secreted [228]. However, ongoing pore formation can permeabilize the infected cell, leading to its osmotic swelling and eventual lysis (reviewed in [224]). *K. pneumoniae* can avoid both efferocytic and pyroptotic clearance by inducing IL-10 expression in infected cells. This provides both an anti-apoptotic signal and inhibits inflammasome activation, thereby limiting both apoptotic and pyroptotic cell death [229]. It is important to note that while PtdSer recognition is involved in the clearance of necrotic and pyroptotic cells, these cell death pathways are highly inflammatory and therefore engulfment of these cells likely does not have the same anti-inflammatory effects as efferocytosis does [230].

#### 7.1.4. *Staphylococcus aureus*: Cell- Specific Manipulation of Apoptosis and Efferocytosis

*S. aureus* is a gram-positive bacterium whose infection of hosts can cause CAP and a host of other diseases involving colonization of different organ systems [231]. While most *S. aureus* is phagocytically cleared by neutrophils, a subset remains viable within the human neutrophil’s phagosomes. The still-viable *S. aureus* induces upregulation of “don’t-eat-me” signal CD47 on the surface of infected neutrophils, thus inhibiting internalization by efferocytes and promoting necroptosis and bacteria dispersal [232]. Through the action of virulence factors such as alpha toxin, *S. aureus* further inhibits neutrophil efferocytosis by alveolar macrophages in infected lungs, independently of CD47 upregulation, by altering infected neutrophils’ expression and localization of the apoptotic cell opsonin CCN1 and the “eat me” signal DD1α [233]. This is in contrast to *S. aureus*-infected macrophages, where *S. aureus* is a potent inducer of apoptosis. Apoptosis of infected macrophages then accelerates bacterial spread, as additional macrophages will be subsequently infected via the efferocytosis of *S. aureus*-bearing apoptotic cells [234].

#### 7.1.5. *Francisella novicida* and *Bacillus anthracis*: Inhibiting Efferocytic Receptors

*Francisella novicida* is an intracellular Gram-negative bacteria that causes pneumonia characterized by necrotic infiltrates in the lung, particularly in immunocompromised patients [235]. Once *F. novicida* infects a macrophage, the efferocytic receptor CD36 is downregulated via an unknown mechanism, leading to the accumulation of necrotic debris in the lung and continued infection [236]. Another pathogen that inhibits signaling through efferocytic receptors is *Bacillus anthracis*, which has been shown to inhibit the activation and phosphorylation of Rac1 downstream of the MERTK and α_V_β_5_ integrin signaling via its virulence factor edema toxin (Figure 3B [105,237]). Thus, the efferocytic uptake of infected apoptotic cells is inhibited as the requisite actin cytoskeletal rearrangements needed to engulf an apoptotic cell are inhibited. This prolongates infection and increases damage from secondary necrosis.

#### 7.1.6. Altered Efferosome Maturation

Another mechanism used by pathogens to avoid efferocytosis is interfering with efferosome/phagosome maturation, thus allowing the pathogen to grow within the phagocyte. Different pathogens can target different steps of the maturation pathway. Perhaps the most well-studied of these pathogens is *M. tuberculosis,* which survives within macrophages through the action of multiple effectors. Upon inhalation, alveolar macrophages promptly phagocytose *M. tuberculosis*, where *M. tuberculosis* then arrests phagosome maturation at several stages. Through modulating the action of GTPases Rab14 and Rab22, fusion of the nascent phagosome with endosomes is inhibited [238]. In addition, the mannosylated lipoarabinomannan (ManLAM) glycolipid secreted into the host cytoplasm by *M. tuberculosis* inhibits intracellular calcium signaling, preventing activation of Rab5 and its effectors such as EEA1, further inhibiting phagosome/efferosome fusion with endosomes (Figure 3C [239]). Other *M. tuberculosis* effectors such as EsxG, EsxH, and PtPa target different elements in the maturation process [240,241]. EsxG and EsxH both target the endosomal sorting complexes required for transport (ESCRT), inhibiting the transport of phagosomes [240] while the phosphatase PtpA secreted into the host cytoplasm dephosphorylates the HOPS complex (Figure 3C), inhibiting the docking of phagosomes with lysosomes, and directly inhibits phagosome acidification by blocking V-ATPase pump assembly [241,242]. Other pathogens, such as *L. pneumophila* and *S. enterica*, target phagosome-lysosome fusion as well through the activity of their virulence factors (reviewed in [243]). Since phagocytosis and efferocytosis share similar maturation mechanisms, the effectors produced by these pathogens to evade microbicidal destruction of phagosome maturation may also allow for their survival following efferocytosis of infected cells.

### 7.2. Manipulating Efferocytosis

While some pathogens have evolved strategies to evade efferocytic elimination, others may exploit host efferocytic mechanisms for their own replication and spread. Through a process termed “apoptotic mimicry”, certain enveloped viruses such as HIV-1, Ebola, Dengue, and vaccinia acquire PtdSer in their viral envelope by budding from PtdSer-rich organelles. The presence of PtdSer in their membranes then allows for these viruses to bind to efferocytic receptor expressing cells, facilitating viral entry [244,245]. Using this strategy, viruses avoid the host’s pro-inflammatory innate and adaptive immune responses, and instead activate the anti-inflammatory signaling cascades of efferocytosis. The parasite *Leishmania major* utilizes a similar mechanism of immune evasion by infecting neutrophils, inducing apoptosis, and gaining entry to the healthy efferocytes that are recruited to the apoptotic infected cell [246]. Similarly, the bacterial pathogen *Listeria monocytogenes* secretes the pore-forming toxin listeriolysin O (LLO, Figure 3D) within the cell cytoplasm, resulting in the budding of host-derived *L. monocytogenes* containing vesicles with exposed PtdSer [247]. Subsequent recognition by efferocytic receptors promotes cell-to-cell spread to uninfected efferocytes. Upon infection of a macrophage, *Yersinia pestis*—the cause of the plague—makes use of “find-me” signaling by inducing production of S1P such that, after release from the dying macrophage, new uninfected macrophages can be found in close proximity for subsequent infection [248]. Thus, pathogens can exploit efferocytosis to disseminate and grow in an immunologically silent manner, both within the lung and in other tissues.

## 8. Therapeutic Interventions that Manipulate Efferocytosis

### 8.1. Existing Therapeutics

Currently, there are several therapeutic interventions that have the potential to manipulate efferocytosis during pneumonia and other inflammatory diseases of the lungs. These treatments—statins and glucocorticoids—function to both enhance alveolar macrophage efferocytosis and to restore lung homeostasis through anti-inflammatory effects.

Statins affect efferocytosis through antagonizing the RhoA pathway, which normally suppresses apoptotic cell engulfment. RhoA, a member of the Rho GTPase family, tightly controls efferocytosis [249,250]. To be functional, RhoA must be covalently modified via prenylation, which attaches a lipid moiety required for RhoA recruitment to the plasma membrane [249,251]. Here, RhoA antagonizes the actin reorganization driven by Rac-1, thereby abrogating efferocytic cup formation and the engulfment of apoptotic cells [249,252]. While statins are typically used to reduce circulating cholesterol levels, the immunomodulatory effects of statins are independent of cholesterol, and instead result from inhibition of HMG-CoA reductase—the rate-limiting enzyme in cholesterol synthesis. Normally, HMG-CoA reductase converts HMG-CoA to mevalonate, with mevalonate then used either in the synthesis of cholesterol, or as a precursor for the prenyl moiety that is attached to RhoA [253]. By limiting the synthesis of this moiety, RhoA cannot be prenylated, thereby preventing this negative regulator of efferocytosis from being recruited to the plasma membrane [251,254,255]. As a result, Rac-1 can mediate efferocytic cup formation and apoptotic cell engulfment unopposed by RhoA activity [252,256]. Simvastatin, for example, was observed to inhibit RhoA in murine lungs infused with apoptotic cells, with simvastatin reducing the fibrosis and damage that otherwise follows infusion of apoptotic cells into the airways [256]. Notably, the effects of simvastatin were also shown in alveolar macrophages from human patients with COPD [201]. These studies indicate that statins may enhance alveolar macrophage efferocytosis in an HMG-CoA reductase-dependent manner to restore lung tissue homeostasis following infection and inflammation [252,257]. This phenomenon has been observed in human patients, where during pneumonia there is a strong correlation between the efferocytic capacity of alveolar macrophages and recovery of lung function following pneumonia. In these patients, those on statins fared better than similar patients not on statins, showing improvements in both efferocytic capacity and recovery of lung function [11]. This demonstrates the potential utility of statins for improving efferocytosis and lung function following inflammatory insults.

Similarly, glucocorticoids enhance alveolar macrophage efferocytosis through interactions with the RhoA pathway [13,249]. Glucocorticoids are small lipophilic molecules that exert their actions through binding to intracellular glucocorticoid receptors. Following binding, the glucocorticoid receptor translocates to the nucleus where it acts as a transcription factor. Synthetic glucocorticoids, such as fluticasone, have been shown to increase the apoptotic cell uptake in murine and human alveolar macrophages through a process termed glucocorticoid augmented efferocytosis (GCAE, [12,13]). GCAE has been shown to enhance alveolar macrophage efferocytosis through multiple mechanisms including limiting the activation of the efferocytosis inhibitor RhoA [258], increased efferocytic and scavenger receptor expression [12,259], and upregulation of pro-efferocytic metabolism through increased expression of LXR and PPAR receptors [260]. This includes upregulation of the efferocytic receptor MERTK—the predominant efferocytic receptor in many tissues including the lung [261,262,263,264,265,266]. In humans, the upregulation of MERTK by dexamethasone has been shown to directly enhance the efferocytic capacity of alveolar macrophages [261,267].

While statins and glucocorticoids improve the efferocytic capacity of alveolar macrophage and may therefore aid in the recovery from pneumonia, these agents have potentially adverse side effects. Statins and glucocorticoids are notorious for their immunosuppressive properties, which may lead to adverse effects such as the recurrence of infection. Statins, in addition to their immunosuppressive effect, may also impair other pro-resolving processes, thus delaying the recovery of lung function [268]. Moreover, statins are known to have adverse side effects, especially in organ systems beyond the lungs. For instance, the reduction of mevalonate levels can lead to deficiencies in mevalonate-derived isoprenoids such as coenzyme Q [269]. This decrease in coenzyme Q levels can lead to impairment of the mitochondrial electron transport chain and antioxidant production, leading to issues with cellular respiration and oxygen depletion, and in severe cases, rhabdomyolysis [270,271]. To minimize these off-target effects, inhaled and intratracheally-administered statins were investigated in murine models of asthma [272,273]. The results confirmed that murine lung epithelial cells contained enzymes required to activate statins. Importantly, these results demonstrated few off-target effects in these murine models [272,273]. Notably, the effects of inhaled statins in humans with asthma remains inconclusive [274], necessitating further studies to determine whether the beneficial effects of inhaled and/or intratracheally-administered statins are observed in other diseases, including pneumonia. Therefore, while statins are generally tolerable, these adverse side effects may be life-threatening and therefore may limit the suitability of statins as a drug for modulating efferocytosis during lung infection or inflammation.

Glucocorticoids have been associated with increased risk of infections, including the recurrence of pneumonia in some patients. Previous human studies demonstrated that hydrocortisone was associated with reduced production of inflammatory cytokines, while dexamethasone was associated with reduced antimicrobial activity [14,275]. Notably, inhaled corticosteroids—which are essential therapeutic agents for patients with COPD—were associated with increased cases of CAP. This finding was further illustrated in studies of murine models of pneumonia, where inhaled fluticasone reduced alveolar macrophage destruction of pneumococci, resulting in increased risk of infection and disease [276]. While glucocorticoids may be effective in temporarily enhancing alveolar macrophage efferocytosis following pneumonia and other associated lung diseases [13], they may have long-term adverse effects on immunity, resulting in increased risk of infection.

### 8.2. Novel Therapeutic Targets

Due to the adverse side effects of statins and glucocorticoids, novel therapies for enhancing pulmonary efferocytosis are required. These novel therapeutics should aim to carefully mimic the anti-inflammatory and pro-healing effects of efferocytosis, while seeking to minimize off-target effects such as increased risk of infection. Two efferocytosis-enhancing therapeutic opportunities that meet these criteria are resolvins and anti-CD47 therapy.

Resolvins have been shown to play a significant role in the recovery of lung tissues to homeostasis following disease, in particular RvD1 after *Pseudomonas aeruginosa* infection. Application of exogenous RvD1 reduced both bacterial growth and neutrophil infiltration, in both acute and long-term murine models of pneumonia [173]. While further studies of the therapeutic potential of resolvins are required in humans, these findings suggest that RvD1 may be a plausible therapeutic target in treating bacterial pneumonia due to its role in inhibiting inflammation and enhancing tissue healing in murine lung models [277,278,279,280]. To date, no human clinical trials with resolvins have been reported, but these compounds are well tolerated in mice [281]. Human trials have focused on improving resolvin production in patients by administration of the dietary fatty acids used as substrates for resolvin synthesis; these treatments increase circulating resolvin levels and are generally well tolerated [282].

CD47 is broadly expressed across most cell types where it acts as a “don’t-eat-me” signal via engaging SIRPα on efferocytes [66]. The engagement of SIRPα recruits phosphatases to the efferocyte plasma membrane which antagonizes protein and lipid kinase activity induced by efferocytic receptors [283,284]. The inhibition of efferocytosis by SIRPα signaling is thought to underly many diseases that are characterized by defective efferocytosis [285,286], and as such, anti-CD47 therapy has been explored as a potential therapeutic approach for treating these diseases [287,288]. For example, atherosclerosis—which is characterized by the accumulation of uncleared apoptotic macrophages beneath the heart vasculature—is responsive to anti-CD47 therapy [285], with this therapy reversing the efferocytic defects that arise early in disease [161]. Importantly, anti-CD47 therapy appears to be well-tolerated by patients, suggesting that this treatment may be suitable for many efferocytosis-associated diseases [289,290]. However, anti-CD47 therapy should be used with caution, as both anemia and thrombocytopenia have been observed in human trials of anti-CD47 therapeutics [291]. Anemia and thrombocytopenia following infusion of anti-CD47 is a result of the expression of CD47 on erythrocytes and platelets; on the former, CD47 normally acts to prevent the efferocytic clearance of non-senescent erythrocytes by splenic macrophages, while on the latter, CD47 prevents spontaneous platelet activation. Blockade of CD47 on these cells then allows for their clearance by macrophages, and whether these side effects can be avoided by using inhaled, rather than injected, CD47-targeting therapeutics has not been tested [292,293,294]. In the lung, anti-CD47 therapy is being investigated in murine models of acute (LPS-induced lung injury) and long-term (*E. coli* pneumonia) lung injury [295]. So far, these studies demonstrated that blocking CD47 enhanced innate and adaptive immune responses to lung infection. Furthermore, anti-CD47 therapy improved apoptotic cell clearance and T cell responses in both acute and long-term murine models of lung infections, reducing pulmonary edema and bacteremia [295]. These findings suggest that blocking CD47 may be a plausible therapeutic approach for enhancing lung efferocytosis and, thus, lung recovery from inflammation and infection.

## 9. Conclusions

This review provided an update on the current mechanisms of efferocytosis, a highly organized process that is essential for the phagocytic removal of apoptotic cell bodies following inflammatory diseases, including the inflammation resulting from pneumonia. Efferocytosis plays an important, but poorly understood role in the pathogenesis of pneumonia, with this effect potentially aggravated by pathogens which manipulate the efferocytic system to enhance their survival. While there are many gaps in this research, such as the lack of human studies regarding the therapeutic potential of various efferocytosis-modifying treatments, current literature shows promise in the use of existing and novel therapeutics in enhancing recovery after pneumonia in murine models, with some evidence showing a similar benefit in human patients.

## Data Availability

Data sharing not applicable.
